# A Functional Neuroimaging Meta-Analysis of Self-Related Processing in Schizophrenia

**DOI:** 10.3389/fneur.2019.00990

**Published:** 2019-09-11

**Authors:** Stéphane Potvin, Lydia Gamache, Ovidiu Lungu

**Affiliations:** ^1^Centre de Recherche de l'Institut Universitaire en Santé Mentale de Montréal, Montreal, QC, Canada; ^2^Department of Psychiatry, Faculty of Medicine, University of Montreal, Montreal, QC, Canada; ^3^Department of Psychology, University of Montreal, Montreal, QC, Canada; ^4^Centre de Recherche de l'Institut Universitaire de Gériatrie de Montréal, Montreal, QC, Canada

**Keywords:** schizophrenia, self-processing, fMRI, meta-analysis, anterior cingulate cortex, prefrontal cortex and thalamus

## Abstract

**Background:** Schizophrenia is characterized by self-disturbances, including impaired self-evaluation abilities and source monitoring. The cortical midline structures (e.g., medial prefrontal cortex, anterior and posterior cingulate cortex, and precuneus) and the temporoparietal junction are known to play a key role in self-related processing. In theory, self-disturbances in schizophrenia may arise from impaired activity in these regions. We performed a functional neuroimaging meta-analysis to verify this hypothesis.

**Methods:** A literature search was performed with PubMed and Google Scholar to identify functional neuroimaging studies examining the neural correlates of self-processing in schizophrenia, using self-other or source monitoring paradigms. Fourteen studies were retrieved, involving 245 patients and 201 controls. Using peak coordinates to recreate an effect-size map of contrast results, a standard random-effects variance weighted meta-analysis for each voxel was performed with the *Seed-based d Mapping* software.

**Results:** During self-processing, decreased activations were observed in schizophrenia patients relative to controls in the bilateral thalamus and the left dorsal anterior cingulate cortex (dACC) and dorso-medial prefrontal cortex. Importantly, results were homogeneous across studies, and no publication bias was observed. Sensitivity analyses revealed that results were replicable in 93–100% of studies.

**Conclusion:** The current results partially support the hypothesized impaired activity of cortical midline brain regions in schizophrenia during self-processing. Decreased activations were observed in the dACC and dorsomedial prefrontal cortex, which are involved in cognitive control and/or salience attribution, as well as decision-making, respectively. These alterations may compromise patients' ability to direct their attention toward themselves and/or others and to make the decision whether a certain trait applies to one's self or to someone else. In addition, decreased activations were observed in the thalamus, which is not a core region of the default-mode network, and is involved in information integration. These thalamic alterations may compromise self-coherence in schizophrenia.

## Introduction

Self-disturbances have been described as core phenotypic features of schizophrenia in the early conceptualizations of the disorder (1, 2). More recently, the importance of self-disturbances has been highlighted by phenomenological investigations (3, 4). Self-disturbances in schizophrenia include a lack of insight into the disorder, an impaired ability to evaluate one's own personal qualities, to identify the source of one's own thoughts and actions, as well as the presence of anomalous subjective experiences (e.g., depersonalization and derealization) (4–9). Since self-disturbances are both prominent and diverse in schizophrenia, some investigators now conceptualize schizophrenia as a meta-cognitive disorder (10). Despite this increasing clinical evidence, the neural bases of self-disturbances in schizophrenia are not yet well-understood. This is due to the fact that, on the one hand, the neural correlates of self-processing in healthy individuals have been well-characterized only in the last decade and, on the other hand, until recently there was not yet a critical mass of similar studies conducted in patients with schizophrenia.

The concept of self has been addressed by many disciplines, such as philosophy, psychology, anthropology, psychiatry, cognitive sciences, etc., including by the newcomer to the table: the neurosciences. While there is a debate about its characterization across these disciplines, there is a consensus that the self is a multifaceted construct, with components that seem to correspond to distinct processes (11, 12). Within this framework, a general distinction is made between two aspects of self: the self-experience (i.e., self as an experiencing subject, sense of personal agency, etc.) and self-related processing (i.e., self as object of knowledge, evaluation of one's personal characteristics, self-representation, etc.). The investigation of self and its neural substrates in cognitive neuroscience has mainly focused on the latter aspect, the self-processing or self-referencing, primarily because it is difficult to devise experimental paradigms that would properly isolate the experiencing self in action (usually implicit) from the task demands at hand (usually explicit). As such, in the current review we focused primarily on studies that focused on the self-processing or self-referencing and operationalized it as being the evaluation of information, such as personal traits, adjectives, statements, etc. in terms of being characteristic to “self” (vs. “non-self” or “others”).

In healthy participants, the neural correlates of self-processing were mostly investigated using self-referencing or source monitoring tasks. In the self-referencing tasks, participants are typically asked to judge whether certain personality traits describe themselves (Self condition) or a significant person (family member, friend, famous person, etc.) (Other condition). Several meta-analyses that included functional magnetic resonance imaging (fMRI) and positron emission tomography (PET) studies conducted in healthy participants using self-referencing tasks showed that cortical midline structures, such as the medial prefrontal cortex (PFC), the anterior cingulate cortex (ACC), the posterior cingulate cortex (PCC) and the precuneus, were significantly activated during the Self condition, relative to the baseline condition (i.e., making lexical or semantic judgments) (13–15). In addition to these structures, consistent activations in the anterior insula and temporo-parietal junction (TPJ) were also observed (13, 16, 17). In several literature reviews parsing out the functional roles of some of these brain regions (18–21), the authors proposed that during processing of self-related information, the ACC would be involved in the allocation of attention toward one's self, while the PCC would be involved in the retrieval of autobiographic memory, the (anterior) insula—in the embodiment of self-experiences, the ventro-medial PFC—in the emotional tagging of self-relevant information, and finally, the dorso-medial PFC (dmPFC)—involved in making the decision whether a certain trait applies to one's self or to someone else. In the case of the TPJ (e.g., posterior superior temporal gyrus and ventral areas of the inferior parietal lobule, including the angular gyrus), it is not only involved in self-processing but is also well-known to play a key role in theory of mind (social cognition) (16, 17, 22, 23). As such, the TPJ has been proposed to be a key mediator between self and other perspectives (24, 25). In addition to self-referencing, source monitoring tasks have also been employed to investigate the neural correlates of self-processing in humans, although less frequently. In these tasks, participants are typically asked to determine the particular origin (self vs. other) of a series of stimuli (verbal or visual) that were generated prior to performing the task. In healthy participants, the patterns of activations observed during self-other source monitoring are similar to those observed in studies using self-referencing paradigms. Indeed, activations in cortical midline structures (e.g., mPFC, ACC, and PCC/precuneus) have been consistently observed during (verbal) source monitoring tasks (26, 27). One potential difference between both paradigms is that source monitoring tasks seem to elicit more temporal cortex activations than self-referencing tasks (28).

Converging evidence indicate that the same cortical midline structures (e.g., mPFC, ACC and PCC) reported to be activated during self-referencing and source monitoring tasks also overlap with brain regions that are typically part of the default mode network as identified in resting-state fMRI studies (9, 29, 30). When participants are scanned in task-free conditions (i.e., resting state), it has been consistently shown that the low-frequency fluctuations in spontaneous brain activity of the mPFC, ACC, PCC, and precuneus are positively correlated with each other over time (29, 30). Since participants are presumably involved, at rest, in mind wandering and introspection, the default mode network is conceptualized as the main neural network involved in self-referential processes (30). While there is an overlap in brain activity between regions involved in explicit self-reference (task-elicited) and implicit self-reference (at rest), there are also differences. Indeed, in two experiments comparing both conditions (31), the authors found that implicit and explicit self-reference commonly engaged the ventral mPFC and PCC, while the dorsal mPFC was preferentially recruited during explicit self-reference, and the precuneus, during implicit self-reference.

Recent meta-analyses of the resting-state fMRI literature in schizophrenia have shown that the connectivity within default mode network is reduced in schizophrenia patients as compared to their healthy counterparts, and, more importantly, that the hypo-connectivity within this network is possibly more prominent in schizophrenia, relative to other psychiatric conditions such as major depressive, bipolar, substance use, and anxiety disorders (32–34).

In contrast to this vast literature on functional connectivity at rest in schizophrenia, it is striking to observe that no meta-analysis has been performed to date on studies investigating the self-related brain activations in this population. Whereas, resting-state fMRI studies are advantageous in that they are more simple to implement by circumventing the problem of task design optimization, classic task-based activation studies are advantageous in that they allow to relate more directly fMRI findings to psychological constructs. However, only in recent years the number of fMRI studies conducted in schizophrenia patients using self-processing tasks have reached the critical mass needed for a meta-analysis on this topic [e.g., (5, 35–40)]. At first glance, these studies have reported abnormal activations during self-processing in schizophrenia in the same cortical midline structures that are typically found in healthy participants; however, the pattern of results is not always consistent since most studies showed reduced activations (patients vs. controls), but a few reported the opposite result. Moreover, altered activations have been reported in some cases in regions unrelated to the default mode network (e.g., insula, temporal cortex, and thalamus), but their significance remains unclear at the moment. Given this heterogeneity and these particularities, a meta-analysis on the neural bases of self-processing in schizophrenia relative to healthy controls is critically needed.

Here, we sought to address this knowledge gap and perform a functional neuroimaging meta-analysis on self-processing in schizophrenia. Given that schizophrenia is associated with self-disturbances, that self-processing tasks recruit activations in cortical midline structures and that the connectivity within the default mode network is reduced in schizophrenia, we hypothesized that decreased activations in cortical midline structures and the TPJ will be observed in schizophrenia patients, relative to healthy participants.

## Methods

### Selection Procedures: Search Strategies

The article search was conducted by two researchers (SP and OL), independently, using PubMed, Google Scholar and Web of Science databases. The search used the following syntax [schizophrenia AND (self OR self-reference OR insight) AND (fMRI OR neuroimaging OR functional magnetic resonance imaging)] and was limited to all original articles (i.e., excluding abstracts from conference proceedings) published before September 20, 2018. It is worth noting that the search syntax is very general and it does not exclude, *a priori*, studies that may investigate self-agency. However, as stated previously, our goal is to identify studies that assessed the functional brain activity underlying the self-processing or self-referencing (and not necessarily the self-agency or the experiencing self) in schizophrenia. A cross-referencing method was also used by manually examining reference lists of the articles included in the meta-analysis.

### Selection Criteria

Studies were included in the meta-analysis provided that they met the following criteria: (i) included a self-reference, self-other distinction, memory source (i.e., self-generated) or insight task (and not self-agency), (ii) contained primary data, (iii) included both psychosis participants (e.g., patients with a diagnosis of schizophrenia-spectrum or psychotic disorder, or participants at risk of psychosis) and a healthy control group and (iv) compared directly the brain activation of these two groups in experimental conditions that included reference to self. Studies were reviewed by two researchers (OL, SP) and inclusion criteria were evaluated by consensus. To achieve a high reporting standard, we followed the “*Preferred Reporting Items for Systematic Reviews and Meta-Analyses*” (PRISMA) guidelines (41) (for more information, see Table 1).

**Table 1:** Description of studies included in the meta-analysis (*N* = 14).

**References**	**N patient group**	**N healthy controls**	**Mean age patients**	**Mean age controls**	**Type of analysis**	**Type of task**	**Task modality**	**Software**	**Magnetic field strength**	**Smoothing (FWHM)**	**TR**
Bedford et al. (42)	11	8	39.0	31.0	WB	Trait judgement	Visual - W	XBAM	1.5	8	2,000
Blackwood et al. (35)	8	8	38.0	36.0	WB	Statement judgement	Visual - W	SPM99	1.5	10	3,000
Holt et al. (36)	18	17	35.9	40.0	WB	Trait judgement	Visual - W	SPM2	3	6	3,000
Jimenez et al. (43)	20	16	48.3	44.7	WB	Trait judgement	Visual - W	FSL	3	5	2,500
Liu et al. (5)	15	15	50.0	40.5	WB	Self-referential task	Visual - W	SPM	3	6	1,500
Makowski et al. (44)	15	15	33.1	35.2	WB	Social approval task	Visual - W	SPM8	3	8	2,000
Menon et al. (37)	14	15	40.5	35.9	WB and ROI	Statement judgement	Visual - W	SPM5	1.5	8	2,300
Murphy et al. (45)	11	10	26.7	29.6	WB	Trait judgement	Visual - W	SPM2	4	6	2,000
Park et al. (46)	14	15	29.5	28.2	WB	Self-referential task	Visual - V	AFNI	1.5	8	3,000
Pauly et al. (39)	13	13	36.2	34.5	WB	Trait judgement	Visual - W	SPM5	3	8	2,400
Sapara et al. (38)	26	16	34.5	31.8	WB	Self-monitoring	Auditory	SPM	1.5	10	3,250
Shad et al. (47)	17	15	40.0	44.3	WB	Self-awareness	Visual - W	SPM5	3	8	2,000
Tan et al. (48)	18	17	40.5	41.2	WB	Trait judgement	Visual - W	SPM8	2	8	3,000
van der Meer et al. (40)	47	21	34.3	30.0	WB	Statement judgement	Visual - W	SPM2	3	10	2,000

*WB, whole brain; ROI, region-of-interest. Task modality: visual (visual stimuli, usually words—W or videos—V, presented on the screen), auditory (auditory stimuli, usually speech, presented binaurally)*.

### Recorded Variables

The variables included in the present meta-analysis, for each article, were: sample size, mean age of patients, antipsychotic dosage (e.g., chlorpromazine equivalents), level of psychiatric symptoms, magnet intensity, voxel size, and repetition time (TR) of functional volumes. Smoothing kernel size was also recorded in the meta-analysis, as recent research has shown this preprocessing parameter is a source of heterogeneity of results in neuroimaging studies (49).

### Meta-Analysis

The meta-analysis was performed using the *Effect-size Seed-based d Mapping* (formerly *Signed Differential Mapping*) (ES-SDM) software (50). The voxel-based approach of ES-SDM is based on the use of *t*-values of peak coordinates to recreate, for each study, an effect-size map of contrast results. To do so, we first extracted peak coordinates and t-statistics of clusters showing significant differences in brain activity at the whole-brain level between schizophrenia patients and healthy volunteers. Both the “schizophrenia > controls” and “controls > schizophrenia” contrasts were used. When the authors reported z-scores instead of t-statistics, these were converted to *t*-values using the t-calculator provided by ES-SDM (http://www.sdmproject.com/utilities/?show=Statistics). Coordinates presented in Talairach space were converted to Montreal Neurological Institute (MNI) space during analysis in ES-SDM. Importantly, studies reporting no statistically significant between-group differences were also included in the meta-analysis. Finally, effect-size brain maps were created by means of an anisotropic Gaussian kernel. Studies were then combined using a random effects model, which takes into account sample size and heterogeneity across studies. Default ES-SDM kernel size and thresholds were used (FWHM = 20 mm, voxel *P* = 0.005, peak height *Z* = 1, cluster extent = 10 voxels) (50).

Robustness of the significant results was assessed by means of exploration of the residual heterogeneity, jack-knife, and subgroup analyses. Publication bias were assessed by examining Egger's test (51) for asymmetry of the funnel plots (52). Jack-knife sensitivity analyses consisted of repeating the meta-analysis iteratively by removing one study at a time to assess the replicability of the results (50). Subgroup analyses were conducted on task contrast (self vs. control; self vs. other) as well as on the smoothing kernel used (5–10 mm^3^). Finally, a meta-regression was performed on mean age of patients, voxel size and TR across studies. The influence of antipsychotic dosage and psychiatric symptoms could not be assessed, as data was available in fewer than 10 studies. Following previous meta-analyses, we increased the probability threshold to minimize the detection of spurious results [see (50) and (53) for further details on robustness analyses].

## Results

### Number of Studies Retrieved

**Figure 1:**
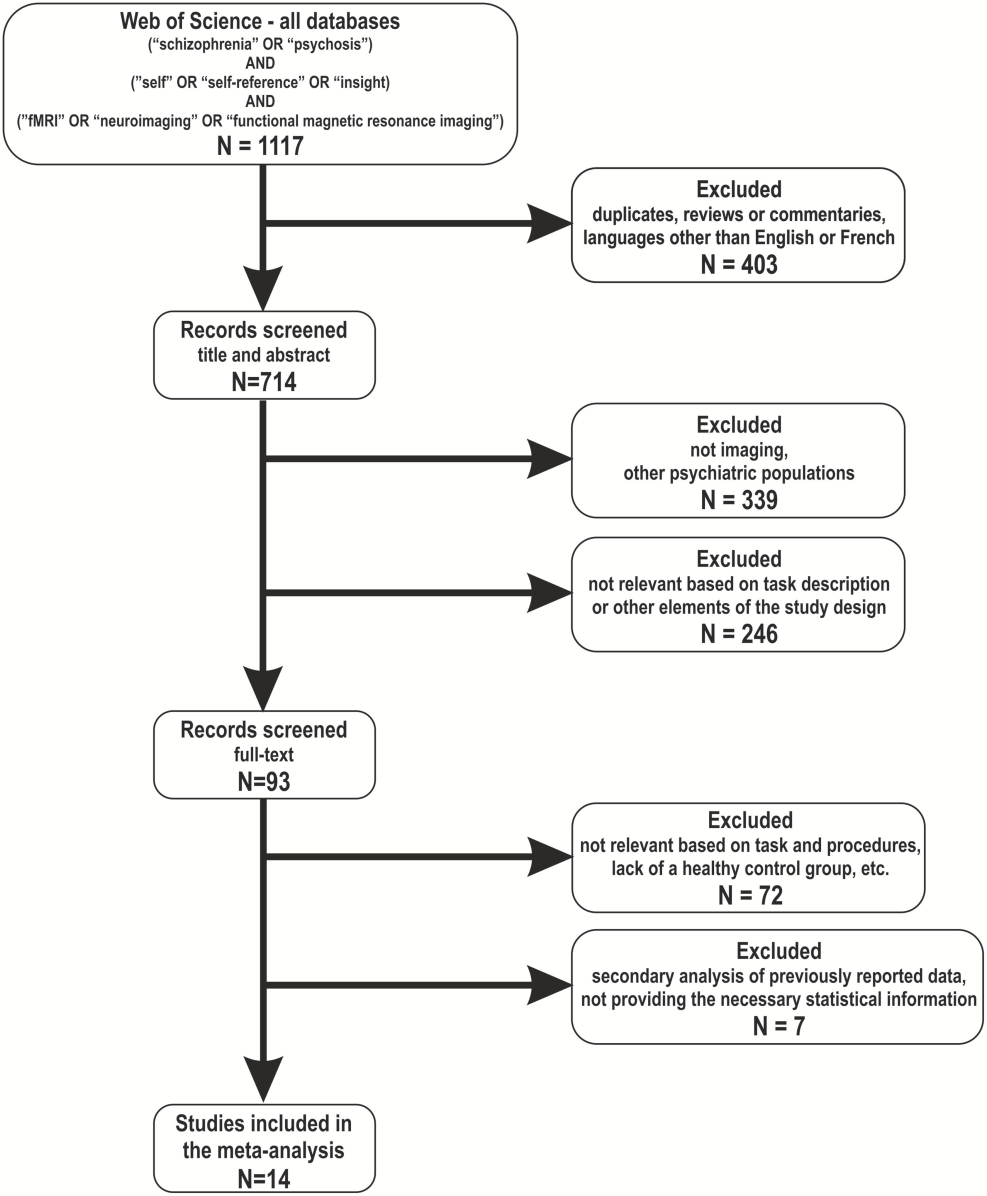
Flow chart of the articles included in the meta-analysis.

After removing duplicates, the initial search yielded 884 articles (as of 20 September, 2018). Of these, 20 studies met the inclusion criteria (i), (ii), and (iv). Of this group, 4 studies were further excluded because it involved individuals at risk for psychosis and not actual patients, and one other study was excluded (54) because it seemed to report data on the same healthy control group and a sub-sample of schizophrenia patients that was included in a previous study, already included in the selection (55). In the course of data analysis, the study from Vinogradov et al. (56) was also excluded, due to its outlier results (defined as 2 standard deviations above or below the composite effect size). A total of 14 studies were included in the final meta-analysis (5, 35–40, 42–48) (see Figure 1 for the flow chart), which comprised a total of 245 schizophrenia-spectrum patients (mean age: 37.5 year) and 201 healthy volunteers. Noteworthy, all included studies used a whole-brain analysis. Eight studies reported results for the Self vs. Other contrast, 4 studies reported results for the Self vs. Control contrast and two studies reported results for both contrasts. Thus, the Self vs. Other contrast results are based on data from 10 studies and those for Self vs. Control—on data from 6 studies. The weighted mean of symptoms level, as measured with the *Positive and Negative Syndrome Scale* total score, was 61.9 ± 10 (*n* = 8 studies). Please refer to Table 1 for details on the included studies.

### Between-Group Differences in Brain Activations

**Figure 2:**
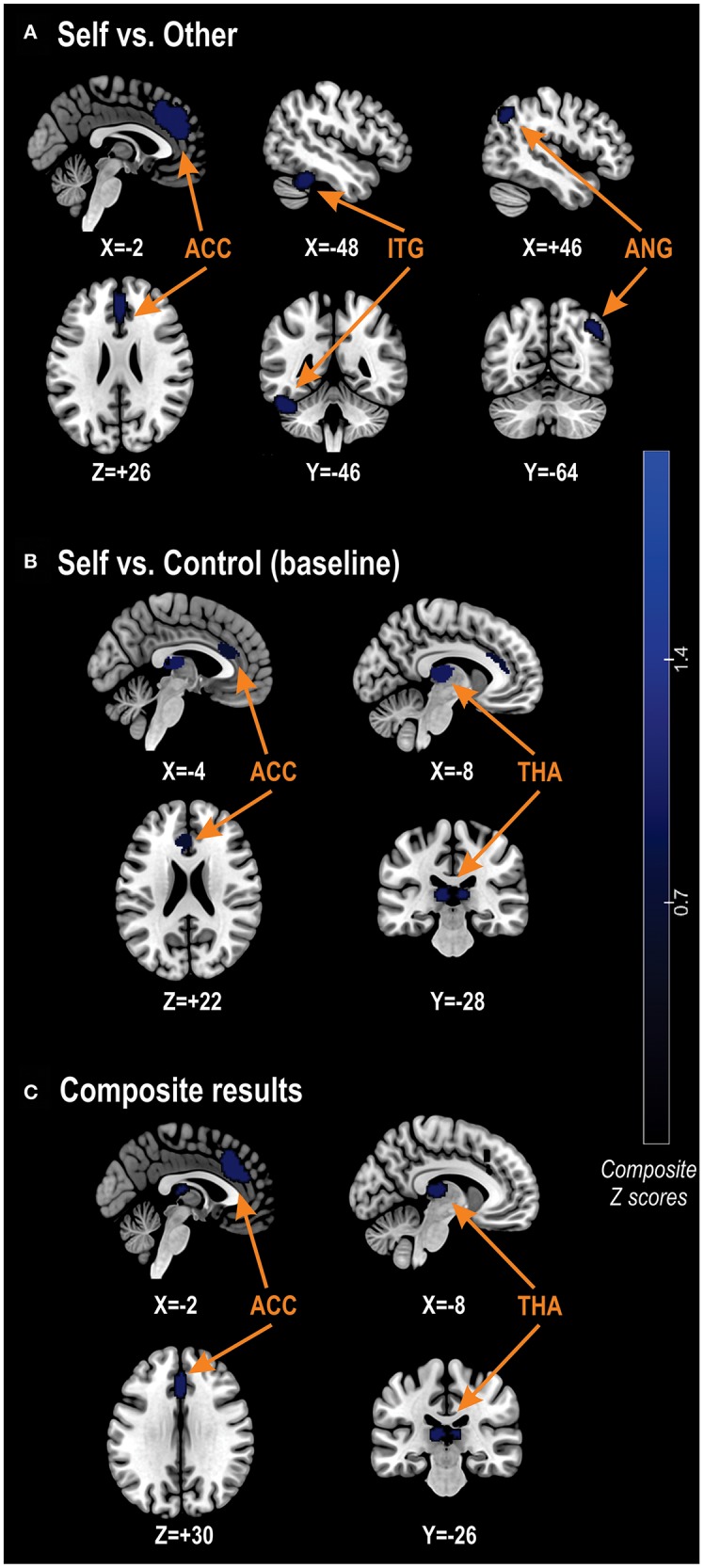
Between-group differences in self-related brain activations. **(A)** Results for the between-group differences in brain activations using the Self vs. Other contrast. **(B)** Results using the Self vs. Control (baseline) contrast. **(C)** Results for the composite analysis, combining every studies including in the meta-analysis. ACC, anterior cingulate cortex; ANG, angular gyrus; ITG, inferior temporal gyrus; THA, thalamus.

For the composite analysis (14 studies), we found that schizophrenia patients had *decreased* activations, relative to controls, in a cluster encompassing the left medial superior frontal gyrus, the left ACC, the bilateral median cingulate cortex, the right superior frontal gyrus, and another cluster encompassing the bilateral thalamus. For these reduced activations in schizophrenia, we observed no significant residual heterogeneity between studies (t = 0.0; *Q* = 9.1; *p* = 0.521). There were no significant *increased* activations in schizophrenia patients as compared to controls for this analysis (Table 2, Figure 2).

**Table 2:** Meta-analysis of brain activations during self-processing in schizophrenia patients relative to controls.

**Region (peak)**	**MNI coordinates**	***Z-*value**	***P*-value**	**No of Voxels**	Breakdown (number of voxels)^**^
COMPOSITE ANALYSIS^*^					
Left anterior cingulate cortex (BA32)	−2; 32; 30	−1.2	0.0009	562	- Left medial superior frontal gyrus (BA32/8, 149), left anterior cingulate cortex (BA24/32, 160), left median cingulate cortex (BA24, 62), right median cingulate cortex (BA32/24, 77), and right superior frontal gyrus (BA32, 24)
Left thalamus	−8; −26; 10	−1.6	~0	265	- Left thalamus (140), and right thalamus (49)
**SELF vs. OTHER CONTRAST**					
Left anterior cingulate cortex (BA32)	−2; 40; 26	−1.4	0.0004	1,073	- Left medial superior frontal gyrus (BA32/9/8, 526), right superior frontal gyrus (BA9, 81), left anterior cingulate gyrus (BA32/24, 153), right median cingulate gyrus (BA32, 54), left median cingulate gyrus (BA24, 44), right anterior cingulate (BA32, 39), and right median cingulate gyrus (BA24, 33)
Left inferior temporal gyrus (BA37)	−48; −46; −28	−1.3	0.001	355	- Left cerebellum, crus I (BA37, 132), left inferior temporal gyrus (BA20/37, 118), left cerebellum, hemispheric lobule VI (BA37, 51), and left fusiform gyrus (BA37, 31)
Right angular gyrus (BA39)	46; −64; 40	−1.2	0.002	205	- Right angular gyrus (BA39/7, 184)
**SELF vs. CONTROL (OR BASELINE) CONTRAST**					
Left thalamus	−8; −28; 10	−1.9	0.00005	573	- Left thalamus (292), and right thalamus (133)
Left anterior cingulate cortex (BA24)	−8; 26; 22	−1.5	0.0005	257	- Left anterior cingulate gyrus (BA24/32, 168)

*BA, Brodmann area; MNI, Montreal Neurologic Institute*.

* *Two studies reported results for both the Self vs. Other and the Self vs. Control contrasts; in these two cases, for the composite analysis, we used the self vs. other contrast, as it was the most frequently employed in the set of studies included in the meta-analysis*;

** *>20 voxels*.

The analyses of robustness (Jacknife analyses) revealed that results were highly replicable since the reduced activations in schizophrenia in the left thalamic cluster were found in 100% of studies, while the reduced activations in schizophrenia in the left anterior cingulate cluster were found in 92.9% of studies (Table 3).

**Table 3:** Jacknife analyses.

**Jacknife analysis**	**Reduced activations in schizophrenia**	
	**Anterior cingulate cortex**	**Thalamus**
Without Bedford et al. (42)	Yes	Yes
Without Blackwood et al. (35)	Yes	Yes
Without Holt et al. (36)	Yes	Yes
Without Jimenez et al. (43)	Yes	Yes
Without Liu et al. (5)	Yes	Yes
Without Makowski et al. (42)	Yes	Yes
Without Menon et al. (37)	*No*	Yes
Without Murphy et al. (45)	Yes	Yes
Without Park et al. (46)	Yes	Yes
Without Pauly et al. (39)	Yes	Yes
Without Sapara et al. (38)	Yes	Yes
Without Shad et al. (47)	Yes	Yes
Without Tan et al. (48)	Yes	Yes
Without Van der Meer et al. (40)	Yes	Yes
**Total**	**13/14**	**14/14**

Finally, in the case of both clusters, no publication bias was detected (left ACC: bias = −1.54; *t* = −1.27; *df* = 13; *p* = 0.227; left thalamus: bias = −1.62; *t* = −0.97; *df* = 13; *p* = 0.351).

#### Meta-Regression Sub-analyses

Using the data from the composite analysis, we found no association between results in the ACC across studies and age (slope = 0.002; *z* = 0.101; *p* = 0.920), temporal resolution (TR; slope = 0.00004; *z* = 0.226; *p* = 0.821), spatial resolution (voxel size; slope = −0.004; *z* = −0.726; *p* = 0.468), and smoothing level (slope = −0.02; *z* = −0.184; *p* = 0.854). Likewise, thalamic results across studies were not influenced by age (slope:0.018; *z* = 1.138; *p* = 0.255), temporal resolution (TR; slope = −0.0004; *z* = −2.341; *p* = 0.019), spatial resolution (voxel size; slope = −0.003; *z* = −0.471; *p* = 0.638) and smoothing level (slope = −0.099; *z* = −1.693; *p* = 0.091).

#### Task Contrasts

For the analysis restricted to the Self vs. Other contrast (10 studies), we found that schizophrenia patients had *decreased* activations, relative to controls, in the left medial superior frontal gyrus, the right superior frontal gyrus, the bilateral ACC, the bilateral median cingulate cortex, the left cerebellum (crus I & hemispheric lobule VI), the left inferior temporal gyrus, the left fusiform gyrus, and the right angular gyrus (Table 2). For this pattern of hypo-activations in schizophrenia, we observed no significant residual heterogeneity between studies (t = 0.0; *Q* = 2.6; *p* = 0.765). As before, we did not find any significant *increased* activation in schizophrenia relative to controls (Table 2).

For the analysis restricted to the Self vs. Control (or baseline) contrast (6 studies), we found significant *decreased* activations in schizophrenia patients relative to controls in the bilateral thalamus, and left ACC (Table 2). There was no significant residual heterogeneity between studies (t = 0.0; *Q* = 2.0; *p* = 0.918) in regards to this result. Again, schizophrenia patients had no *increased* activations relative to controls (Table 2).

## Discussion

To our knowledge, this is the first functional neuroimaging meta-analysis to examine the neural correlates of self-processing in schizophrenia. Fourteen studies were retrieved, and the aggregation of their results showed that activations in the dorsal anterior cingulate cortex (dACC), the dorsomedial prefrontal cortex (dmPFC) and the thalamus display reduced activations in schizophrenia patients, relative to healthy participants, during self-processing. Importantly, the results of the meta-analysis were homogeneous and robust, and were not influenced by publication biases. In secondary analyses, we found that patients' age, as well as neuroimaging parameters (e.g., temporal resolution, spatial resolution, and smoothing level) had no influence on results.

Comparing our findings with those reported in the previous meta-analyses on the neural bases of self-processing in healthy individuals (13–15) we observe that, on the one hand, we have found hypo-activations only in a core node region involved in self-referential processing in healthy controls (dmPFC) and in a region involved in cognitive control and/or salience attribution (e.g., dACC) and, on the other hand, we have localized a group difference in a subcortical region outside the typical self-processing network (thalamus). Considering that all 14 studies included in the current meta-analysis reported results of brain-wise contrasts (i.e., not restricted to previously reported regions of interest), the localization of the hypo-activations in two of the self-referential midline cortical structures indicate a high construct validity. Moreover, given the critical role of thalamus as a gateway relaying of sensory and motor information, and regulating consciousness, mood, sleep and alertness (57–60), the reduced activation found in this region is consistent with the recently proposed view that self-related impairments seen in schizophrenia patients may be a reflection of a fragmented self, due to the isolation and reduced modularity of brain networks involved in intrinsic and extrinsic self-processing (9).

Given that the dACC and dmPFC are core cortical midline structures, the reduced activations found in these regions in schizophrenia provide support for the main hypothesis that we sought to test in the current meta-analysis. The dACC is inter-connected with frontal, striatal, and limbic regions, and its roles are complex and matter to debate. Nevertheless, there is ample evidence from task-based fMRI studies showing that the dACC and the adjacent median cingulate cortex play a key role in cognitive control and attention (21, 61). In complementary fashion, a vast resting-state functional connectivity literature has shown that the dACC is one of two core nodes of the salience network (the other being the anterior insula) (62, 63). In schizophrenia, several studies have shown that the connectivity between the dACC and the anterior insula is reduced (33). In theory, it has been proposed that the salience network is involved in the orientation of attention to the most salient internal and external stimuli. Accordingly, it may be argued that the reduced dACC activations observed in schizophrenia patients may compromise their ability to allocate or shift their attention toward themselves and/or others. As for the dmPFC, Van der Meer et al. (13) proposed in their model that this region would be involved in the *decision making* processes involved in self-referencing tasks. If so, the reduced dmPFC activations observed in schizophrenia may indicate that these patients experience difficulties in deciding whether a certain personality trait applies to one's self or to someone else. However, the results of a recent neuroimaging meta-analysis from Eickhoff et al. of fMRI studies performed in healthy participants on the roles of the dmPFC suggest that a slightly different interpretation of our results is possible (64). Indeed, this meta-analysis has highlighted that the dmPFC and the PCC are significantly co-activated, and that the dmPFC plays a key role in social cognition, noticeably *theory of mind* (64). The results of the meta-analysis from Eickhoff et al. (64) suggest that the reduced dmPFC activations observed in schizophrenia may not reflect impaired decision making abilities *per se*, but rather a difficulty in attributing mental states to others. Interestingly, in the current meta-analysis, we found that the dmPFC activity was reduced in schizophrenia only in the fMRI studies using a “self vs. other” contrast, whereas the studies using a “self vs. control/baseline” contrast showed no between-group differences in dmPFC activity. In that regard, it is interesting to note that a neuroimaging meta-analysis from Kronbichler et al. on social cognition showed that schizophrenia patients have reduced dmPFC activations while performing theory of mind tasks (65).

While the reduced dACC and dmPFC activations found in schizophrenia during self-processing are generally consistent with main hypothesis of the current meta-analysis, we also found that schizophrenia patients had reduced activations in the thalamus, which is not considered a cortical midline structure, but rather being part of a network involved in information gating (33) or as part of the salience network (cortico-striato-thalamo-cortical)—the mediodorsal thalamus (66). This finding is particularly novel in that the vast majority of investigators who performed the fMRI studies on self-processing in schizophrenia in the current meta-analysis highlighted the importance of alterations in cortical midline structures (5, 36, 37, 39, 40, 42–45, 47, 48). Conversely, we are not aware of any investigator who discussed the importance of the thalamus, meaning that the role of this region has clearly been neglected thus far. Noteworthy, thalamic activity was found to be decreased only in studies using a “self vs. baseline/control” task contrast. The thalamus is massively interconnected with the whole cerebral cortex, the cerebellum, and is involved in information integration from every sensory system (67), information gating (33), as well as in awakening and consciousness (68). As such, the thalamic alterations found in schizophrenia during self-processing are possibly indicative of a lack of self-coherence. The observed thalamic alterations observed here are coherent with the fact that the thalamus is growingly considered as being critically involved in the pathophysiology of schizophrenia. Indeed, data from the ENIGMA consortium has shown in 2,028 schizophrenia patients and 2,540 healthy controls that schizophrenia is associated with a small to moderate (*d* = 0.31) decrease in thalamic volumes (69). Likewise, several resting-state functional connectivity studies have shown that the connectivity between the thalamus and frontal, cingulate, sensorimotor, and cerebellar regions is significantly reduced in schizophrenia (70, 71). Perhaps more importantly, a recent multi-modal neuroimaging meta-analysis of resting-state functional connectivity studies and voxel-based morphometry studies showed that among all the regions found to be impaired in schizophrenia patients relative to healthy controls, the thalamus was one of the few regions found to be not only reduced in volumes, but more prominently impaired in its functional connectivity in schizophrenia patients, relative to patients with bipolar, major depressive, substance use and anxiety disorders (32).

Apart from the dmPFC, dACC, and thalamus, other regions were found to be significantly impaired in schizophrenia during self-processing. Noticeably, reduced activations in the right angular gyrus were observed in schizophrenia patients relative to controls. In addition to the cortical midline structures (e.g., mPFC, ACC, PCC, and precuneus), the angular gyrus is one of the core regions of the default mode network (29, 30). The right angular gyrus is part of the TPJ and is involved in several functions, including self-processing, spatial cognition, attention, and theory of mind (17, 22). The involvement of the right angular gyrus in theory of mind is of particular interest in the current context. Indeed, the reduced activations observed in this brain region in schizophrenia were only observed in the case of the studies using a “self vs. other” contrast, but not in the studies using a “self vs. baseline/control” contrast. This result is consistent with the notion that the TPJ is involved the mediation between self and other perspectives. As such, the result suggests that self/other differentiation is impaired in schizophrenia, and the TPJ is involved in this impairment. Finally, in the studies using a “self vs. other” contrast, reduced activations were also observed in schizophrenia in a cluster encompassing the left inferior temporal cortex, fusiform gyrus, and cerebellum. While the inferior temporal cortex is involved in color, face and object recognition, and semantic memory, the fusiform gyrus is involved in face and object recognition and reading, and the cerebellum crus I is primarily involved in higher cognitive functions (72–74). In view of these heterogeneous functions, it is difficult to interpret the reduced activations observed in this cluster in schizophrenia during self-processing. However, two of these regions (inferior temporal cortex and fusiform gyrus) are located along the inferior longitudinal fasciculus and are part of the ventral visual stream. Previous studies have shown that the integrity of the inferior longitudinal fasciculus was associated with semantic (as opposed to episodic) autobiographical memory (75), that ventral stream increased activation was associated with successful encoding of emotional information (76). In light of these previous findings, our results could be interpreted as a functional deficit in the processing of emotional autobiographical memories in schizophrenia patients. The fact that the cluster included also the left Crus I/lobule VI of the cerebellum strengthens this interpretation. Indeed, a quantitative review of cerebellar findings in fMRI literature reported not only that cerebellar hypoactivations predominate across studies and various tasks in schizophrenia, but that emotional tasks yielded results in the left lobule VI (77).

In recent years, there is increasing evidence that the cerebellum plays a broader role in cognition than previously thought (78), thus challenging the traditional view that it is primarily involved in motor control (79). Indeed, a recent review of neuroimaging and clinical studies highlights the cerebellar involvement in performance monitoring across a variety of domains and tasks (80) and suggesting that monitoring may be cerebellum's “overarching function” (81). This view is consistent with the findings from recent meta-analyses on the role of cerebellum in social cognition, which identified “mentalizing networks” within the cerebellum that are active when people engage in self-judgement or self-processing tasks (82–84). Given the findings from the meta-analysis by Van Overwalle et al. (82) that have identified activation clusters in left lobule VI when mentalizing about distant others and activation in Crus I when performing abstract mentalizing tasks, the cerebellar hypoactivation in these regions in our meta-analysis seems to indicate that the deficit in these kind of self-processing tasks in schizophrenia may be due to the malfunctioning of these mentalizing networks. As such, the cognitive dysmetria hypothesis in schizophrenia and its cerebellar substrate may be expanded to include self-processing.

The current meta-analysis suffers from a few limitations that need to be acknowledged. First, schizophrenia patients were treated with antipsychotics at the moment of being scanned, meaning that we cannot determine if our results are related to schizophrenia, to antipsychotic medication and/or to a combination of both factors. Antipsychotics are known for blocking dopamine release in the associative striatum (85). Other than that, the impacts of antipsychotics on brain structure and function remain unclear. Thus far, the majority of studies have paid attention to the anatomical effects of antipsychotics (86). As for the fMRI studies, the available evidence tends to show that antipsychotics *normalize*, rather than impair, task-based activations and resting-state functional connectivity in schizophrenia (87). Moreover, little evidence shows that antipsychotics have beneficial effects particularly on the functioning of the brain regions of the default mode network (87). Still, in the pool of studies included in our meta-analysis, not enough of them reported the mean antipsychotic dosage (e.g., chlorpromazine equivalents) of patients, so we were not able to perform a meta-regression analysis, which would have allowed to investigate the potential influence of antipsychotic medication on the abnormal activations observed in schizophrenia during self-processing. Likewise, not enough studies reported the level of symptoms of patients to perform meta-regression analyses. In the past, some authors proposed that self-disturbances in schizophrenia may be related to the positive symptoms of the disorder (6), while others proposed that the lack of insight of some patients is due to self-disturbances (38). Unfortunately, in the current meta-analysis, we were not able to examine both possibilities. Moreover, there were not enough studies to perform sub-analyses on the type of task used in the scanner (e.g., trait judgment vs. source monitoring). Also, we did not contact authors, so statistical maps were not used for analysis, and this may have limited statistical power. Finally, we were not able to include the studies involving individuals at risk for psychosis in the meta-analytic analyses, since only 4 studies were identified, despite the fact that heterogeneous definitions of psychosis risk were considered (e.g., schizotypy, first-degree relatives, siblings) (88–91). Thus far, 3 of these studies have shown altered activations in cortical midline structures (e.g., dmPFC and PCC) during self-processing in individuals at risk for psychosis relative to typically developing individuals.

The current results partially support the hypothesized impaired activity of cortical midline structures in schizophrenia during self-processing. Indeed, decreased activations were observed not only in cortical midline structures (e.g., dACC and dmPFC), but also in the thalamus, which is not a core region of the default-mode network. Taken together, the results of the current meta-analysis suggest that self-disturbances in schizophrenia are related to decreased activity in brain regions involved in attention and/or salience attribution (e.g., dACC), decision-making and/or theory of mind (e.g., dmPFC), as well as experiential coherence (e.g., thalamus). Future enquiries in the field will need to combine analyses of resting-state functional connectivity and analyses of task-based activations, as they provide complementary information. An important issue will be to investigate the functional connectivity between the default-mode and salience networks during explicit self-processing in schizophrenia, similar to the emerging investigations on the interactions between these networks at rest in this population (92). In the future studies on the topic, careful attention will need to be paid to the potential impact of antipsychotic medication. Finally, more studies are warranted on the neural alterations associated with self-disturbances in individuals at clinical or biological risk for psychosis.

## Author Contributions

SP conceived the idea of the study, did the search of studies, performed the meta-analytic statistics, and wrote the manuscript. OL did the search of studies, performed the data extraction, prepared the tables and figures, and wrote the manuscript. LG was involved in the study selection, did the data extraction, prepared the tables and figures, prepared the references, and provided the detailed critical comments. All authors approved the final version of the manuscript.

### Conflict of Interest Statement

The authors declare that the research was conducted in the absence of any commercial or financial relationships that could be construed as a potential conflict of interest.
